# Age-Related Differences in Contribution of Rule-Based Thinking toward Moral Evaluations

**DOI:** 10.3389/fpsyg.2017.00597

**Published:** 2017-04-20

**Authors:** Simona C. S. Caravita, Lindamulage N. De Silva, Vera Pagani, Barbara Colombo, Alessandro Antonietti

**Affiliations:** ^1^Department of Psychology, Centro di Ricerca sulle Dinamiche Evolutive ed Educative (CRIdee), Università Cattolicà del Sacro CuoreMilan, Italy; ^2^Department of Psychology, Center for Research on Vocational Guidance and Socio-Professional Development, Università Cattolica del Sacro CuoreMilan, Italy; ^3^Education and Human Studies, Champlain CollegeBurlington, VT, USA

**Keywords:** moral development, moral reasoning, decision making, social cognition, middle childhood, adolescence, age-related differences, neuroscience

## Abstract

This study aims to investigate the interplay of different criteria of moral evaluation, related to the type of the rule and context characteristics, in moral reasoning of children, early, and late adolescents. Students attending to fourth, seventh, and tenth grade were asked to evaluate the acceptability of rule breaking actions using *ad hoc* scenarios. Results suggest that the role of different moral evaluation criteria changes by age. During adolescence a greater integration of the moral criteria emerged. Moreover, adolescents also prioritized the evaluation of moral rule (forbidding to harm others) violations as non-acceptable when the perpetrator harms an innocent victim by applying a direct personal force. The relevance of these findings to increase the understanding of how moral reasoning changes by age for the assessment of impairments in moral reasoning of non-normative groups is also discussed.

## Introduction

One of the first neuropsychological studies of moral judgment and decision making led Greene et al. (2004) to develop a Dual Process Theory to explain moral decision making. They highlight that human beings can be driven by two distinct modes when they have to provide a behavioral response to an environmental stimulus. The first mode is automatic, based on emotions, reflexes, and intuition; the second is a deliberate mode, and it is useful when reasoned choices, and behaviors based on a specified and detailed knowledge of the surrounding situation are needed. The latter mode is conscious, based on specific rules and it requires more energy expenditure than the first mode. Brain imaging studies reported by Greene et al. (2004) have shown and confirmed that these two modes correspond to different neural activations. Specifically, automatic responses are produced by the ventromedial prefrontal cortex (VMPFC) while controlled and conscious responses from the dorsolateral prefrontal cortex (DLPFC).

When discussing this Dual Process Theory of moral judgment, Greene et al. (2001) argued that *characteristically deontological judgments* (i.e., judgments applied to rights, duties, etc.) are supported by automatic and emotional responses, while *characteristically utilitarian judgments* (which are based on cost-benefit reasoning) tend to be supported by conscious response under the control of cognition (Greene, 2014).

According to some recent literature showing the relevance of the rule-based thinking for moral judgments (Nichols et al., 2016; Ayars and Nichols, 2017), it is noteworthy to highlight that the processes involved in the deontological and utilitarian evaluations may be influenced by the organization of the knowledge about rules in distinct domains. Within the theoretical framework of the Moral Domain Theory (Turiel, 1998) it has been hypothesized that the moral knowledge is structured in separate domains, which are mainly related to the distinction between moral rules, aimed at preserving the other's well-being (e.g., the rules forbidding to hit or kill another person), and socio-conventional rules, aimed at guaranteeing the order of the social system (e.g., the school-based rule forbidding to call the teacher by their first name). There is some evidence that in people's evaluations, violations of moral rules are usually considered universally wrong, independently from the context in which they happened and independently from any statement that can be made by authorities. On the other side, violations of socio-conventional rules are judged as less serious and less punishable if compared to breaking moral rules, because the value of socio-conventional rules is perceived to depend on authorities' statements (Smetana and Braeges, 1990).

The Dual Process Theory was inspired by the “trolley problem,” a moral dilemma widely used for the study of moral reasoning and decision making. The trolley problem describes a situation where a runaway trolley threatens to kill several persons if it proceeds on its present route. In the “switch” (*impersonal*) version of the problem, a bystander can save those persons by flipping a switch that will deviate the trolley to an alternative track, where it will kill one person instead of five. In the “footbridge” (*personal*) version of the dilemma, the bystander stands next to a large stranger on a footbridge that spans the trolley track, and can stop the trolley by pushing the stranger off the bridge on the track below. When asked to face the trolley problem, most people agree that the decision of switching the trolley is acceptable (*characteristically utilitarian judgment*), but they evaluate pushing the stranger off the bridge as impermissible (*characteristically deontological judgment*), even if in both the situations the behavior intentionally causes the death of one person in order to save several people (Greene et al., 2001; Nichols and Mallon, 2006; Bartels, 2008). The switch problem and the footbridge problem are considered, respectively, prototypical examples of *impersonal* and *personal* dilemmas.

The tendency to accept moral rule violations more easily when they are presented in impersonal rather than in personal contexts has been confirmed for all the versions of moral dilemmas that have been developed based on the trolley problem. Moreover, fMRI studies showed that brain areas related to emotions were more activated while examining personal (including the footbridge problem) than impersonal dilemmas (Greene et al., 2004). Neuroscientific studies have also provided plenty evidence that both adults and children tend to accept harming the innocent victim much more when facing an impersonal situation than a personal situation (Greene et al., 2001; Pellizzoni et al., 2010; Greene, 2014). Nevertheless, the reason why personal and impersonal dilemmas elicit activation of different neural networks, and, even more interestingly, elicit different rates of accepting moral violations is still unclear.

A first explanation has been based on the assumption that the core criterion causing this difference in moral evaluations consists in the presence (in personal dilemma contexts) or absence (in the impersonal dilemma contexts) of the application of a personal force between the actor and their sacrificial victim (Greene et al., 2009). This explanation also implies that the presence vs. absence of the application of a personal force, mainly by means of a physical contact, elicits a different sense of harm aversion due to reduced empathic concern for the victim (e.g., Hauser et al., 2007; Patil and Silani, 2014). Following this line of reasoning, the presence of the application of a personal force in the interaction between the actor and the victim may elicit empathic feelings in the person evaluating the action, because the proximity will foster a personal identification with the actor. This explanation, however, fails to explain some of the research results reported in other studies that used the original series of dilemmas. These conflicting results lead to hypothesize that other criteria are mixed with the personal force criterion (Greene et al., 2001, 2009). In particular, the most relevant variable to play an effect seems to be linked to the harm of the innocent person being or not being the result of deflecting an existing threat onto a different party. Therefore, there is still a need to test the personal force criterion in a rigorous and controlled way, in order to be able to clarify whether it can actually be considered a main criterion influencing moral evaluations. In order to do so, the experimental design should control all confounding variables, by the way of having the presence or absence of a personal force applied by the agent to the victim as the only systematic variation distinguishing between personal and impersonal contexts of the moral violations. Furthermore, there is the need to test the personal force criterion by means of dilemmas that, in comparison to the trolley dilemma, describe situations closer to the everyday people's experience, and that, therefore, are more ecological.

### A model of the possible interplay of moral evaluation criteria

Within the debate on moral judgment in the utilitarian framework of personal and impersonal situations, Nichols and Mallon (2006) proposed that three factors play a role in evaluating the permissibility of moral rule violations: (1) cost/benefit analysis, (2) checking for rule violations and prioritizing the rule value, and (3) emotional activation. When deciding about acceptability of behaviors, people usually examine costs and benefits for each line of actions and check whether those actions break rules, in particular the moral rule that forbids to harm others. Concerning the third factor, Nichols and Mallon (2006) hypothesized that the features of the context can elicit different levels of emotional activation that, in turn, influence the two cognitive processes of cost/benefit analysis and evaluation of the moral rule. Specifically, personal situations would elicit higher emotional activation in comparison to impersonal situations, because the application of personal force by the perpetrator would prompt empathizing with the victim. As a consequence, when personal situations are presented, the evaluation of the action as respectful/transgressive of moral rules becomes a priority above the cost-benefit analysis. On the other hand, when the perpetrator's action is performed in absence of the application of a personal force (i.e., impersonal context), in the moral evaluations the consideration of how much the action is beneficial predominates on checking for moral rule violations. This model can help explaining why violations of moral rules can be judged as admissible under some context conditions, and it suggests that personal and impersonal dilemmas may provide a critical test of the relative relevance and weight of the processes involved in forming the decision to perform a social action (Nichols and Mallon, 2006). When facing personal dilemmas, we can expect that checking for rule violations is prompted by the presence of the application of a personal force that is typical of the perpetrator's action and, therefore, that this process will win over the cost/benefit analysis and lead to consider the action as non-permissible. On the other hand, when presented with impersonal dilemmas, in which the application of a personal force in the perpetrator's action is absent, the process of analyzing the costs and benefits of the action becomes prominent and lead to evaluate harming others as acceptable.

### Developmental differences in moral evaluations

Up to now, the research within the framework of the Dual Process Theory has been focusing mainly on the assessments of moral violations in personal and impersonal dilemmas based on the trolley problem presented above and using adult samples. More research involving different age-groups and based on more ecological situations is still needed.

To our knowledge, only few studies focused on children (Pellizzoni et al., 2010; Caravita et al., 2012; Powell et al., 2012; Cushman et al., 2013; Margoni and Surian, 2017). In three experiments focusing on preschoolers' evaluations of the trolley problem (Pellizzoni et al., 2010), most of 3- to 5-year old children judged as a right choice to hurt one person in order to save five, but only when it did not require the agent to have a physical contact with the victim (the switch version of the dilemma), thus resembling adults' answers. Accordingly, Powell et al. (2012) found that children aged 5–6 and 7–8 years, when facing situations that mirrored the personal and the impersonal dilemmas, produced evaluations similar to adults' ones.

Nevertheless, assuming an adult model of moral reasoning as a universal model could potentially be misleading, and hence investigating better possible changes in the weight of different criteria in determining moral evaluations has been recommended (Nichols and Mallon, 2006). In order to achieve this goal, we need (1) to further explore possible age-related differences in the dual process moral reasoning by comparing different age-groups, and (2) to consider a greater number of personal and impersonal situations, which should be more ecological.

When focusing on the rule-based thinking, studies designed within the framework of the moral domain theory have investigated this process from a developmental perspective. Within this perspective, the research has provided evidence that by the early age of 34 months children are able to distinguish between basic moral and socio-conventional events. By 42 months of age children are also able to judge a moral violation as more serious and non-admissible than a socio-conventional violation, and they perceive the violation of the moral as wrong independently of authorities' statements and rule contingency (Smetana and Braeges, 1990). Starting at the age of 3 years, children are also aware of the consequences of actions, and they judge behaviors that break moral rules as wrong because intrinsically unfair (Helwig et al., 2001).

Within this line of research, most of the existing studies have focused on preschoolers. Nevertheless, the little research involving older samples of children and early adolescents has provided evidence that the ability of distinguishing between domains continues to increase by age (Glassman and Zan, 1995). Younger children have been found to consider moral rules as dependent on authority's statements (thus, as socio-conventional) at a larger extent than older children do (Caravita et al., 2009; Gasser and Keller, 2009), and, when evaluating a behavior in situations of mixed domains, preschoolers and first graders tend to evaluate these behavior as socio-conventional more than third graders do (Crane and Tisak, 1995).

Age-related changes within the general domain structure, however, need to be further explored by considering a larger range of situations. Indeed, most of the existing studies focused on events where the target transgressive action involved only physical harm (Smetana, 2006) and studies on the moral domain distinction during late-childhood (8–9 years) and adolescence, that is, the age periods that are traditionally assumed as critical for the development of rule socio-conventionality (Kohlberg, 1981; for a review, Killen and Smetana, 2006), are scarce.

### The present study

The first goal of this study is exploring how children and adolescents, when engaged in moral reasoning, use the criteria of moral judgment that the Dual Process Theory individuates as relevant for the moral evaluations. We mainly focused on the contribution of rule-based thinking, as conceptualized in the framework of the moral domain theory, toward reasoning about moral dilemmas. For this purpose, we referred to the model proposed by Nichols and Mallon (2006). This model provides a possible interpretative hypothesis of how the contextual dimension of the presence/absence of a personal force applied by the agent in harming the victim may influence the evaluation of the acceptability of moral rules violations within an utilitarian framework. We hypothesize that the evaluation of moral rule violations as acceptable is more strongly associated to accepting to harm a person in order to benefit others in impersonal than in personal situations. This derives from the assumption that when personal situations are presented, the application of a personal force by the agent in harming the victim prioritizes the respect of moral rules (forbidding to harm others) over the cost/benefit analysis. In contrast, in impersonal situations, where there is no application of personal force by the agent, the cost-benefit analysis often prevails over the respect of moral rules, and the moral rule violation is more easily accepted.

The second purpose of the study is exploring age-related differences in how children and adolescents integrate the moral evaluation criteria that were individuated by the Dual Process Theory and by the moral domain theory (adopted as theoretical model of how the rule-based thinking is organized). This would foster a better understanding of how different criteria of evaluation interact, and affect moral decision making at different ages, within the normative population. This information may foster a better understanding of how moral reasoning develops, and help to better identify possible impairments in non-normative groups. To achieve this goal, we compared three age levels that are considered to be critical for the development of rule understanding and moral reasoning (Killen and Smetana, 2006): a sample of 8–9 year children to 12–13 years early-adolescents and to 15–16 years adolescents. As far as moral reasoning in personal/impersonal contexts is concerned, we hypothesize that all 3 age groups will accept moral violations more easily in impersonal rather than personal situations, reflecting a universal trend. Assuming a developmental perspective, a task coherent with the domain theory (Loureno, 2014) would have been the most appropriate to use. To achieve this goal, an ecologically valid battery of moral dilemmas, suitable for the target population, was developed.

Because this is the first study assuming that the rule-based thinking is organized in different domains and exploring how this form of thinking works in association with the cost-benefit analysis in different age-groups, we can only formulate preliminary hypotheses about age-related differences in the associations between evaluation criteria in judging violations of moral vs. socio-conventional rules, and violations of the moral rules in personal vs. impersonal contexts. We hypothesize that the integration between evaluations of violations of both moral and socio-conventional rules and of moral rule violations in personal and impersonal contexts increases by age, because of the development of cognitive processes.

## Methods

### Participants

The study involved 226 students attending two primary schools, two middle schools, and two high schools in the areas of Milan and Brescia, two of the largest cities in Northern Italy. At the time of the data collection, 81 participants were in fourth grade (primary school group: 59.3% boys; *M* = 8.98 years; *SD* = 0.39), 72 were in seventh grade (middle school group: 55.6%% boys; *M* = 12.14 years; *SD* = 0.61), and 73 were in tenth grade (high school group: 52.1%% boys; *M* = 15.10 years; *SD* = 0.38). Most (81.9 %) participants were born in Italy and of Italian lineage.

Information on the SES of participants' families was collected by asking participants to report their parents or legal caregivers' qualifications and jobs. Only 2 participants (0.9%) were not able to provide this information. Twenty-one percent resulted of low-middle SES, 56.3% of middle SES, and 22.8% of middle-high SES.

### Measures

#### Moral vs. socio-conventional domains

A specific measure was used to evaluate domain attribution to rules and acceptance of social violations based on distinguishing between moral and socio-conventional domains. The assessment tool (Caravita et al., 2012) was composed by 21 items, consisting in short stories where a moral school rule was broken, and 20 items in which a socio-conventional school rule was violated. All the items required the respondent to assume the perspective of the character breaking the rule. In half of the scenarios characters were girls and in half boys. Two versions of the measure were created: one for children and one for early-adolescents and adolescents. The two versions were equivalent for the content, the word number, and the grammar structure of the items. They differed only for contextual factors, which were related to primary school in the children version and to secondary school in the adolescent version (in the Italian school system middle and high school share the same main contextual characteristics). After each item was presented, the respondent was asked to judge whether the main character's behavior in that situation was right (*acceptability of rule violations*). Samples items are displayed in Table 1.

**Table 1:** **Examples of moral and socio-conventional scenarios**.

**Condition**	**Moral scenario**	**Socio-conventional scenario**
Text description	In your school there is a rule that you must not take other children's things. A day at school you are in the cafeteria and you force John to give you his lunch and then you eat it. (*Primary school scenario*)	In your school there is the rule that children must stand up when an adult enters the classroom. One morning a janitress enters into the classroom and you don't stand up because you are drawing. (*Middle school scenario*) In your school there is a rule forbidding to leave the books out of the personal lockers, at the end of the lessons. One morning, you are in a hurry to leave school and you abandon your history book on your desk. (*High school scenario*)
Acceptability of rule violations	Is it right to do so? (Yes = 1; No = 0)	

*Each scenario was followed by a question on the Acceptability of rule violation*.

For both moral and socio-conventional scenarios, the total score of the acceptability of rule violation is computed as the sum of the item scores. Reliability of scores (Cronbach's alpha) was from acceptable to good for the children version (moral rule scenarios 0.67; socio-conventional rule scenarios 0.83) and good for the adolescent version (moral rule scenarios 0.91; socio-conventional rule scenarios 0.90).

#### Personal vs. impersonal moral dilemmas

Eight couples of impersonal and personal dilemmas, that is, 16 dilemmas altogether, to be administered to primary school children, and a corresponding series of dilemmas to be administered to elder students were used (Caravita et al., 2012). The characters acting in the dilemmas were all children, girls in half of the dilemmas and boys in the second half. Each dilemma presented a scenario where a “helpmate,” in order to produce a benefit to another child, has to act so that a harm, a loss, or a disadvantage affects a third child (henceforth the “victimized child”). For each of the eight scenarios there was an impersonal and a personal version. Being the contextual information the same, in impersonal scenarios there was no physical contact between the helpmate and the victimized child, that is, the helpmate did not interact face to face with the victim; In personal scenarios the helpmate touched, looked at, and/or spoke to the victimized child. Presence/absence of physical contact between the helpmate and the victimized child was the unique, unequivocal criterion that distinguished between the personal and the impersonal dilemma versions and possible confounding variables were discarded (Antonietti, 2011).

Focusing on formal features, dilemmas consisted of short stories, easy to be understood, not involving the knowledge of specific social norms, not describing dramatic and emotionally impressive situations. The victimized child was always unaware of the situation (otherwise she/he might decide to sacrifice her-/himself) and could not avoid being involved in the action carried out by the helpmate. The helpmate could not sacrifice her-/himself instead of the victimized child. The action performed by the helpmate always had a certain outcome. The helpmate took no direct advantage or damage as a consequence of her/his action.

Two series of dilemmas were prepared for children (i.e., the primary school students) and for adolescents. The two series only differed from each other for situation details, which matched what usually occurs in everyday-life school experiences of, respectively, children and elder youths. Examples of pairs of dilemmas are displayed in Table 2.

**Table 2:** **Examples of personal and impersonal scenarios**.

**Condition**	**Personal scenario**	**Impersonal scenario**
Text description	Dario is tumbling down the stairs. Alberto is in front of you at the bottom of the stairs. You push Alberto so that he falls on his knees and stops the fall of Dario, and Dario does not hurt himself too much. (*Primary, Middle and High school*)	Andrea is tumbling down the stairs. Luca is in front of you. You pushes a pile of paper boxes that is on the stairs so that they can stop Andrea's falling, even if they knock Luca down too. (*Primary, Middle and High school*).
Acceptability of rule violations	Is it right to do so? (Yes = 1; No = 0)	

*Each scenario was followed by a question on the Acceptability of rule violation*.

Each dilemma ended with the question: “Is it right to do so?” (Yes = 1; No = 0). Total scores of personal and impersonal dilemmas were the sum of dilemma responses, with higher scores corresponding to accepting hurting the victimized child. Reliability (Cronbach's alpha) of the sub-series of dilemmas was acceptable in both the versions, but better for the adolescent version (personal dilemmas 0.70; impersonal dilemmas 0.72) than for the children version (personal dilemmas 0.55; impersonal dilemmas 0.50).

### Procedure

A broader study was carried out in collaboration with the rehabilitation center IRCCS “Eugenio Medea” (Bosisio Parini, Italy), in order to include atypically developing participants. The ethical committee of the IRCCS “Eugenio Medea” approved the whole research project, including the current study, which only involved typically developing participants. Written informed consent for all participants was collected in accordance with the Declaration of Helsinki.

Personal and impersonal dilemmas were mixed and then divided into two sequences so that the personal and impersonal versions of the same dilemma were not included in the same sequence. The moral and socio-conventional scenarios were mixed as well and then organized in two sequences. The four sequences were then alternated and their order was reversed, thus obtaining two administration protocols. Protocol 1 started with one of the two sequences of moral and socio-conventional stories, whereas in protocol 2 the starting sequence was one of the two dilemma sequences. In each school-level half of the classes answered the protocol 1 and the other half the protocol 2.

Measures were group-administered in participants' classrooms during normal school time in a single session (approximately 90 min). A research assistant supervised the administration, gave information about filling out the measures, answered participants' questions, and in primary school classes read aloud each item of the measures, giving the children enough time to answer. Class participation was authorized by principals and committees of teachers. Participants' parents (or legal guardians) were informed of the study aims and procedure by means of a letter that was sent by the schools. Parents actively consented the children's participation by signing the consent form attached to the letter. At the beginning of the session, participants were informed about the aims of the research project and their right to withdraw from the study at any time, and were asked for a verbal consent to their participation. Only 5 students (2.19% of the sample) were not allowed to participate in the study.

## Results

### Associations of moral components

Correlations (Pearson's *r*) of the moral evaluation criteria for the overall sample are reported in Table 3.

**Table 3:** **Overall sample: Correlations of moral evaluation criteria (acceptability of the rule violations)**.

	**1**.	**2**.	**3**.	**4**.
1.Moral rules	–			
2.Socio-conv. Rules	0.72^***^	–		
3.Personal context	0.32^***^	0.37^***^	–	
4.Impersonal context	0.47^***^	0.45^***^	0.60^***^	–

^*^p < 0.05, ^**^p < 0.01,

*** *p < 0.001*.

Accepting the moral rule violation in personal and impersonal contexts was positively associated with judging rule violations as acceptable for both moral and socio-conventional rules. Correlations in the separate age groups are reported in Table 4.

**Table 4:** **Correlations of moral evaluation criteria (acceptability of the rule violations) in the separate age-groups**.

	**1**.	**2**.	**3**.	**4**.
**CHILDREN**				
1.Moral rules	–			
2.Socio-conv. rules	0.63^***^	–		
3.Personal context	0.19	0.35^**^	–	
4.Impersonal context	0.15	0.30^**^	0.54^***^	–
**EARLY ADOLESCENTS**				
1.Moral rules	–			
2. Socio-conv. rules	0.74^***^	–		
3.Personal context	0.27^*^	0.39^***^	–	
4.Impersonal context	0.50^***^	0.55^***^	0.73^***^	–
**ADOLESCENTS**				
1.Moral rules	–			
2. Socio-conv. rules	0.69^***^	–		
3.Personal context	0.46^***^	0.44^***^	–	
4.Impersonal context	0.59^***^	0.48^***^	0.51^***^	–

* *p < 0.05*,

** *p < 0.01*,

*** *p < 0.001*.

Among children the levels of acceptability of violations of moral and socio-conventional rules were highly positively associated to each other. The same was true for accepting the moral rule violations in personal and impersonal contexts. However, the acceptance of harming a person in both personal and impersonal contexts was connected to the acceptance of violations of socio-conventional rules but not of moral rules.

Among early adolescents, accepting rule violations in both the domains was positively correlated with accepting to harm others in impersonal and personal contexts, but the correlation was stronger for the impersonal than the personal dilemmas. These differences in the strength of the correlation indices were significant at the Steiger's z test: correlations of accepting breaking moral rules with personal/impersonal scenarios, *z* = −2.906, *p* < 0.01; correlations of accepting breaking socio-conventional rules with personal/impersonal scenarios, *z* = −2.115, *p* < 0.05. Also, among adolescents, accepting violation of both moral and socio-conventional rules was more strongly associated with accepting to harm others in impersonal than in personal contexts, but these differences in the strength of the associations were not significant at the Steiger's *z*-test (moral rules: *z* = −1.365, *ns*; socio-conventional rules: *z* = −0.394, *ns*).

### Age-related trends of moral evaluation criteria

In order to investigate age-related trends, data were first analyzed by performing repeated-measure Analyses of Variance (ANOVA) by assuming scores in the series of moral vs. socio-conventional rule scenarios and personal vs. impersonal dilemmas as dependent variables, type of the rule/context as within-subject factor, and age-group and gender as between-subject independent variables. Two separate ANOVAs (Tables 5, 6) were, therefore, carried out: one for acceptability of violations of moral and socio-conventional rules and one for personal/impersonal contexts.

**Table 5:** **Means, standard deviations of moral and socio-conventional rule scenarios, and indexes of the ANOVAs tests**.

	**Moral rule Scenario**		**Socio-conv. Rule Scenario**	
	***M***	***SD***	***M***	***SD***
Children	0.46	1.09	1.70	2.62
Boys	0.64	1.31	2.00	2.90
Girls	0.21	0.60	1.27	2.11
Early adolescents	2.00	3.56	4.36	5.08
Boys	2.38	3.64	4.75	5.07
Girls	1.53	3.45	3.88	5.12
Adolescents	2.71	3.93	6.80	5.15
Boys	3.89	4.38	7.68	5.37
Girls	1.43	2.93	5.83	4.78
Total	1.68	3.21	4.20	4.85
Boys	2.18	3.51	4.61	5.03
Girls	1.06	2.67	3.70	4.59
Type of rule/context	*F*_(1, 219)_ 142.23^***^, η^2^ = 0.394			
Age-groups	*F*_(2, 219)_ 22.23^***^, η^2^ = 0.169			
Gender	*F*_(1, 219)_ 6.89^**^, η^2^ = 0.030			
Type of rule/context X Age	*F*_(2, 219)_ 15.53^***^, η^2^ = 0.124			
Type of rule/context X Gender	*F*_(1, 219)_ 0.05 *ns*, η^2^ = 0.000			
Age X Gender	*F*_(1, 219)_ 1.14 *ns*, η^2^ = 0.010			
Type of rule/context X Age X Gender	*F*_(2, 219)_ 0.40 *ns*, η^2^ = 0.004			

^*^p < 0.05,

** *p < 0.01*,

*** *p < 0.001*.

**Table 6:** **Means, standard deviations of the personal and impersonal scenarios, and indexes of the ANOVAs tests**.

	**Personal context**		**Impersonal context**	
	***M***	***SD***	***M***	***SD***
Children	1.60	1.42	2.23	1.60
Boys	1.71	1.44	2.56	1.62
Girls	1.43	1.41	1.73	1.46
Early adolescents	2.02	1.63	2.68	1.87
Boys	2.32	1.58	3.05	1.86
Girls	1.61	1.64	2.18	1.81
Adolescents	1.79	1.45	2.53	1.78
Boys	2.14	1.38	2.64	1.82
Girls	1.41	1.46	2.41	1.74
Total	1.79	1.50	2.47	1.75
Boys	2.03	1.48	2.74	1.76
Girls	1.48	1.49	2.12	1.68
Type of rule/context	*F*_(1, 219)_ 41.51^***^, η^2^ = 0.168			
Age-groups	*F*_(2, 219)_ 1.63 *ns*, η^2^ = 0.016			
Gender	*F*_(1, 219)_ 9.25^**^, η^2^ = 0.043			
Type of rule/context X Age	*F*_(2, 219)_ 0.26 *ns*, η^2^ = 0.003			
Type of rule/context X Gender	*F*_(1, 219)_ 0.12 *ns*, η^2^ = 0.001			
Age X Gender	*F*_(1, 219)_ 0.22 *ns*, η^2^ = 0.002			
Type of rule/context X Age X Gender	*F*_(2, 219)_ 2.29 *ns*, η^2^ = 0.022			

*p < 0.05,

** *p < 0.01*,

*** *p < 0.001*.

**Figure 1:**
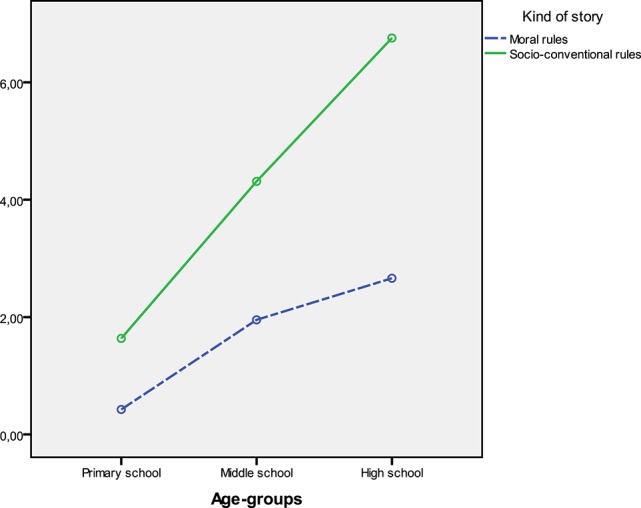
**Mean scores of the 3 age-groups for acceptability of moral and socio-conventional rule violations**.

Concerning the acceptability of rule violations in moral vs. socio-conventional scenarios, main effects of gender, type of rule, age-group, and the interaction effect of type of rule X age-group emerged. Girls evaluated rules as less breakable than boys. Acceptance of rule violations increased by age. Violations of rules were evaluated as less acceptable for moral rules than socio-conventional rules by all participants. However, the difference in scores between moral and socio-conventional rules gradually increased across age-groups (Figure 1). Univariate ANOVAs performed in order to better investigate the interaction effect of type of rule X age-group showed that scores for accepting the rule violations were lower for the moral rules than for the socio-conventional rules in all the three age-groups [Children: *F*_(1, 79)_ = 27.71, *p* < 0.001, η^2^ = 0.26; Early adolescents: *F*_(1, 71)_ = 33.98, *p* < 0.001, η^2^ = 0.32; Adolescents: *F*_(1, 72)_ = 83.94, *p* < 0.001, η^2^ = 0.54]. The three age-groups differed from each other in reference to both moral [*F*_(2, 222)_ = 10.75, *p* < 0.001, η^2^ = 0.09] and socio-conventional rules [*F*_(2, 222)_ = 25.78, *p* < 0.001, η^2^ = 0.19]. Yet, at the Student-Newmann-Keuls *post hoc* test, the scores of the 3 age-groups were all significantly different from each other for the socio-conventional rule violations, but not for the moral rule violations. Concerning the moral rule violations, only the subsample of children scored significantly lower than early adolescents and adolescents.

When considering personal vs. impersonal contexts, the main effect of the type of context was statistically significant, with lower scores for personal as compared to impersonal dilemmas. Gender also had a significant main effect, with girls scoring lower than boys. No significant effects by the age group emerged.

### Effects of the context on accepting moral rule violations

We referred to the Nichols and Mallon's Model by investigating the hypothesis that the evaluation of moral rules as non-breakable is less influential on accepting the harming actions in impersonal contexts than in personal contexts. This would happen because perceiving moral rule as non-breakable is hypothesized to be less prioritized on the cost-benefit analysis in impersonal situations than in personal situations (Nichols and Mallon, 2006). This means that accepting the violations of moral rules should be more strongly associated with harming another person in impersonal than in personal contexts.

To test this hypothesis, we performed a repeated measure Analysis of Covariance (ANCOVA) with scores of personal vs. impersonal dilemmas as a within-subject factor. The acceptability of violations of moral rules was specified as a covariate. The expectation was to find a significant interaction effect of the type of dilemma by the acceptability of the moral rule violations. According with our hypothesis, the acceptability of the moral rule violation not only affected scores through the dilemmas [*F*_(1, 209)_ = 47.28, *p* < 0.001, η^2^ = 0.18], but also its interaction effect by the type of dilemma was significant [*F*_(1, 209)_ = 15.28, *p* < 0.001, η^2^ = 0.07]. To better examine the interaction effect, follow-up regressions were performed: Scores of personal and impersonal dilemmas were specified as the criterion variables and the acceptability of the moral rule violations as the predictor variable.

As hypothesized, the acceptability of the moral rule violations explained a significant portion of variance of scores of both personal [*R*^2^ = 0.10, *F*_(1, 215)_ = 24.57, *p* < 0.001] and impersonal dilemmas [*R*^2^ = 0.22, *F*_(1, 215)_ = 60.74, *p* < 0.001], but its association with the dilemmas score was stronger for the impersonal [β = 0.47, *p* < 0.001] than for the personal contexts [β = 0.32, *p* < 0.001].

In order to explore whether this interplay of the variables changes by age, the ANCOVA test was carried out separately within each age-group. Among children, both the main effect of the acceptability of violations of moral rules and its interaction effect by the type of context were not significant [respectively: *F*_(1, 73)_ = 2.43, *ns*; *F*_(1, 73)_ = 0.09, *ns*]. Among both early adolescents and adolescents, the main effect of the acceptability of moral rule violation was significant [Early adolescents: *F*_(1, 64)_ = 10.96, *p* < 0.01, η^2^ = 0.15; Adolescents: *F*_(1, 68)_ = 40.50, *p* < 0.001, η^2^ = 0.37], and the interaction effect of type of context X acceptability of moral rule violation as well [Early adolescents: *F*_(1, 64)_ = 21.09, *p* < 0.001, η^2^ = 0.25; Adolescents: *F*_(1, 68)_ = 4.18, *p* < 0.05, η^2^ = 0.06]. The follow-up regressions confirmed that in both the age-groups the acceptability of the moral rule violations was associated with accepting to harm a person more strongly in impersonal [Early adolescents: *R*^2^ = 0.25, *F*_(1, 65)_ = 22.16, *p* < 0.001, β = 0.50 *p* < 0.05; Adolescents: *R*^2^ = 0.35, *F*_(1, 68)_ = 37.39, *p* < 0.001, β = 0.59 *p* < 0.001] than in personal dilemmas [Early adolescents: *R*^2^ = 0.08, *F*_(1, 65)_ = 5.56, *p* < 0.05, β = 0.27 *p* < 0.05; Adolescents: *R*^2^ = 0.25, *F*_(1, 68)_ = 17.90, *p* < 0.001, β = 0.46 *p* < 0.001].

As a further test of Nichols and Mallon's Model, we also performed an ANCOVA with scores of personal vs. impersonal dilemmas as a within-subject factor and the acceptability of violations of socio-conventional rules as a covariate. Since personal and impersonal dilemmas describe mainly situations in which moral rather than socio-conventional rules are broken, we can expect that the interaction effect of accepting socio-conventional rule violations with personal vs. impersonal contexts is lower, as compared to the interaction effect of accepting moral rule violations, or non-significant. We tested the ANCOVA separately for the three age groups, since the age level already showed to buffer these effects in the previous analyses. Among children and adolescents the interaction effect of the acceptability of socio-conventional rule violations with type of the dilemma was non-significant [children: *F*_(1, 73)_ = 0.05, *ns*; adolescents: *F*_(1, 68)_ = 1.12, *ns*]. Among early adolescents the interaction effect of acceptability of socio-conventional rule violations with type of the context was significant [*F*_(1, 64)_ = 8.91, *p* < 0.01, η^2^ = 0.12], even if lower than the interaction effect of accepting moral rule violation in the same age group. Likewise acceptability of moral rule violations, in follow-up regression the acceptability of socio-conventional rule violations was associated more strongly with harming the victimized child in impersonal contexts [*R*^2^ = 0.15, *F*_(1, 65)_ = 28.41, *p* < 0.001, β = 0.55 *p* < 0.001] than in personal contexts [*R*^2^ = 0.30, *F*_(1, 69)_ = 12.12, *p* = 0.001, β = 0.39 *p* = 0.001].

## Discussion and conclusions

The first goal of this study was exploring the possible interplay of criteria of moral evaluations reflecting the organization of moral knowledge in domains related to types of rules, and the evaluations of moral violations in personal and impersonal contexts as proposed in the Dual Process Theory (Greene et al., 2004). We assumed the model proposed by Nichols and Mallon (2006) as a possible interpretative hypothesis to understand this interplay. Our second goal was exploring whether the associations of moral evaluation criteria and their interplay in judgments change by age. Possible moderation by age was examined by considering three separate age groups: children, early adolescents, and adolescents.

As far as the first goal is concerned, correlation indexes showed that judging rule violations as acceptable was associated to evaluating harming another person as possible in order to help a third one. This association was stronger for impersonal than personal contexts. The result was significant for the all sample, even if it was more distinctive for the two adolescents groups. These data provide a first confirmation of the potential relevance of context characteristics in prioritizing the check of coherence between evaluations of the behavior and the respect of moral rules over the analysis of costs and benefits *per se* (the model by Nichols and Mallon, 2006). The tendency to permit moral rule violations was associated to accepting the harm of others, if this provides a possible benefit. This association was weaker in the personal contexts, which were characterized by the presence of a personal force applied by the agent in harming the victim, than in the impersonal contexts. Furthermore, the tendency to allow violations of socio-conventional rules had either non-significant or, in comparison to the acceptability of moral rule violations, weaker interaction effect with the presence/absence of the application of personal force by the agents. This result provides some evidence that it is the judgment about moral rules, but not the judgment about socio-conventional rules, that, in moral evaluations, is influenced by these context characteristics, and, hence, it supports the relevance of the rule-based thinking for moral judgements. Therefore, Nichols and Mallon's model that focuses on how different judgment criteria, related to context dimensions and types of rules, interact in influencing the moral evaluations, seems to provide an adequate perspective to better understand how different moral criteria play a role in the moral decision making. This may subsequently foster a clearer understanding of the organization of moral knowledge and reasoning.

An interesting and possibly controversial finding is the positive correlation that emerged between violation of socio-conventional rules and permissibility of harm in impersonal and personal moral dilemmas. It is easy to see that disregard for moral rules can lead to acceptance of harm for the greater good, but it is less clear why disregard for conventional rules is associated with utilitarian moral judgments. What all of these judgments share is the common determination of norm violation. So someone could argue that what our results track using moral domain task is the propensity to abide by norms and this is what is actually predictive of norm violation in a different context. Some evidence from literature may help explaining this specific result. Huebner et al. (2009), exploring the role of emotions in moral evaluations, hypothesize that a fast, unconscious process that operates over causal-intentional representations mediates our moral judgments. Such a fast and unconscious mechanism would clearly explain our results, since it would elicit an answer before the distinction between moral and socio-conventional rules has been fully processed at a cognitive level, pertaining the reading of it to the moral domain and not to a general response to norms.

### Age-related trends in moral evaluations

The distinction between moral domains has been found to increase with age. Coherently with our hypotheses, the understanding of socio-conventional rules as more breakable than moral rules was higher among early adolescents than children, and among adolescents than among younger age groups. Overall, the picture emerging from our data is in line with the traditional view of a gradual improvement, across adolescent years, of the comprehension of the nature of rules and of socio-conventional rules as changeable and based on the social agreement (e.g., Kohlberg, 1981). Accordingly, adolescents become progressively more able to understand the nature of the rules and tend to evaluate the rule violations as more acceptable for the socio-conventional rules than for the moral rules. Nevertheless, surprisingly, primary school children judged moral rules as significantly less breakable than older youngsters. The expectation would have been that the age-level was not influential on the judgment of moral rule violations as not acceptable. However, it should be noted that the size of these two effects was low (0.09 and 0.02) and that this outcome may reflect a view of rules, independent of the type, which is overall more rigid for children than for adolescents. In accordance with this interpretation, children showed the lowest differences in accepting moral and socio-conventional rule violations and revealed a less undifferentiated comprehension of norms.

As we hypothesized, and consistent with previous literature involving other age samples (Pellizzoni et al., 2010), comparing the personal and the impersonal dilemmas, children and adolescents showed the same tendency, already found in adults, to consider harming another person as acceptable more easily in contexts where the agent's application of personal force in harming the victim is absent than in contexts entailing this personal force. It is important to highlight that this tendency emerged in this study using scenarios that were more ecological than the original trolley problem. The apparent universality of this behavior gives some support to theories assuming the existence of an innate moral sense (Sachdeva et al., 2011), or, at least, an innate set of parameters for building morality (Hauser, 2006). Assuming that personal contexts are associated to higher activation of emotion-related brain areas than the impersonal contexts are (Greene et al., 2001), we can hypothesize that emotions may be one of the innate tools for moral intuitions and judgments (Haidt, 2001). This is coherent with the idea that the early distinction between moral and socio-conventional domains is grounded in empathy (Helwig, 2008). Empathic emotions may establish the value of moral rules and the emotional activation elicited by the application of personal force by the harm perpetrator may make the concordance between action and moral rules a priority (Nichols and Mallon, 2006).

When exploring possible age-related differences in the interplay of criteria influencing moral evaluations, the correlation indexes showed that the associations between accepting violations across the four situations increased with age. This outcome suggests an increase of coherence in moral judgment with age, probably reflecting the development of cognitive skills linked to moral reasoning that leads to a more integrated functioning. Accordingly, we found support for Nichols and Mallon's model among early adolescents and adolescents, that is, the two age groups that were expected to show the strongest integration in moral reasoning, but not among children. Similarly, Powell et al. (2012) found that children (5–6 years old) integrated less the cost/benefit analysis in their justifications for allowing harming a person in order to save five than older children (aged 7–8 years) did. We can hence assume that the ability to consider more evaluation criteria in moral situations is likely to increase and to show higher levels of integration with age. These findings and the outcomes on age-related differences for the domain structure emerging from the exam of correlations and ANCOVAs highlight the need to further investigate the relations among different evaluation criteria in moral decision making, and the underlying structure of morality. These criteria potentially influence the moral reasoning to a different extent across different age groups. This interpretation agrees with the literature suggesting that at different ages young people attribute different weights to specific dimensions (such as agent's intention and the respect of the rule) when producing moral evaluations (Helwig et al., 2001), and supports the relevance of exploring how these criteria, studied in different traditions of research, interact and change their interplay according to the child's development. This investigation can provide a better understanding of how moral reasoning changes within the normative population by age, and, subsequently, can provide relevant reference points to evaluate moral reasoning skills and development in non-normative groups of different age.

### Limitations and future directions

There are some limitations in this study. First, the collected data were only cross-sectional. Therefore, we could investigate developmental differences in the organization of moral reasoning only by comparing different age groups. Furthermore, we did not explore the role of emotions in influencing moral reasoning. Hence, we could only hypothesize that differences in emotional activation and emotional response can be also influential on the different weight attributed to different moral evaluation criteria, which are related to specific context characteristics. Future studies can further investigate the possible role played by cultural differences. Literature on this specific topic offers mixed results (Tang and Tang, 2016). On one hand, some form of a “generalized ethic,” only marginally influenced by culture, has been reported. For instance, Mann et al. (2016) showed that dishonesty is limited in magnitude and similar across countries and it is not associated to cultural values. Moreover, Kwan (2016) observed that both American and Chinese participants disapproved the infringement of intellectual property rights to a similar extent and expressed the same level of anger in response to to such a violation. On the other hand, by contrast, cultural factors, as argued by Graham et al. (2016) in their review of the literature, appear to influence moral judgments and moral behaviors, since differences in morality occur not only across societies, but also within the same national context, when comparing inter-societal subgroups. Sychev et al. (2016) found that, according to self-report measures of morality, Mongolian and Russian adolescents share conservative moral foundations, whereas German adolescents exhibit more progressive moral foundations.

With specific reference to moral dilemmas, Arutyunova et al. (2016) proposed tasks similar to the trolley problem, both involving and not involving personal contacts, via Web to a large sample of North American and British people in comparison to Russian participants. They found cultural differences only in the male subsample, with men from Western cultures resulting more utilitarian than Russian men. Focusing on children, Michelin et al. (2010), by using a version of the trolley problems that was adapted for children, reported that in a sample of Italian mono-lingual children the preference for an utilitarian criterion (namely, saving more persons) did not emerge until the age of 6 years. Instead, 4 and 5-year children with a Slovenian-Italian linguistic and cultural background applied such a criterion even in the personal condition. The distinct effect of culture and language should be disentangled, as stressed by the study conducted by Chan et al. (2016), where participants made more utilitarian choices in the Footbridge dilemma when it was presented in a foreign language than in their native language.

As further limitation, the current study only focused on hypothetical judgments. Nevertheless, the actual behavioral responses may change in more realistic contexts, as found in some previuos studies (Patil et al., 2014; Francis et al., 2016). Lastly, reliability of the series of personal and impersonal dilemmas was acceptable but only moderate among children.

Notwithstanding these limitations, there are several novelties in this study. This is one of the first studies exploring the relations between different moral evaluation criteria, and the related organization of moral knowledge in domains and of moral reasoning factors related to specific contextual facets. Moreover, this study is one of the first attempts to join lines of research on moral reasoning that are usually separate in other studies. The value of linking different study traditions has been highlighted as a future promising direction of research (Killen and Smetana, 2008). From the same perspective, we need further research projects exploring from a developmental perspective the interplay of different moral criteria, emotions, and cognition in determining moral evaluations, and the actual moral behavior in the normative population, in order to be able to gain a better understanding of possible impairments and deficits in moral reasoning of non-normative groups, such as children or adolescents with traumatic brain injury (Beauchamp et al., 2013).

As a third novelty of this study, this research project is one of the few studies testing the universality of the distinction between moral judgements for personal and impersonal contexts in non-adult groups and, to our knowledge, this is the first study performing this test using three separate age groups: children, early adolescents, and adolescents.

As a last strength of this study, we used ecological scenarios to test differences between personal and impersonal contexts, and assessed moral domain features using a large selection of scenarios. This research approach can be promising for improving our understanding of the architecture of morality and of its development with age.

## Ethics statement

This study was carried out in accordance with the recommendations of American Psychology Association, and Associazione Italiana di Psicologia. Written informed consent was collected from parents/legal guardians of all participants, in accordance with the Declaration of Helsinki. All participants gave also oral informed consent. The protocol was approved by the ethical committee of the IRCCS “Eugenio Medea” (Bosisio Parini, Italy).

## Author contributions

SC contributed substantially to the conception of the study, the development of the measures, the data collection, the data analyses and the interpretation of the results, and to drafting the article. She also leaded the writing process of the article. LD contributed substantially to the development of the measures, and the data collection. She also contributed to the first draft of the article. VP contributed substantially to the data analyses and the interpretation of the results, and to writing the article. BC contributed substantially to the data analyses and the interpretation of the results, and to writing the article. AA contributed substantially to the conception of the study, the development of the measures, the data collection, the data analyses and the interpretation of the results, and to writing the article. He leads the research of the group on this topic.

## Funding

The publication of the present paper was supported by the Catholic University of the Sacred Heart, Milan, Italy, thanks to a grant delivered within the research funding line D3.1.

### Conflict of interest statement

The authors declare that the research was conducted in the absence of any commercial or financial relationships that could be construed as a potential conflict of interest.
